# Cannabidiol to Improve Mobility in People with Multiple Sclerosis

**DOI:** 10.3389/fneur.2018.00183

**Published:** 2018-03-22

**Authors:** Thorsten Rudroff, Jacob Sosnoff

**Affiliations:** ^1^Department of Health and Exercise Science, Colorado State University, Fort Collins, CO, United States; ^2^Department of Radiology, University of Colorado School of Medicine, Aurora, CO, United States; ^3^Department of Kinesiology and Community Health, University of Illinois at Urbana-Champaign, Champaign, IL, United States

**Keywords:** cannabis, multiple sclerosis, pain, spasticity, inflammation

Multiple sclerosis (MS) is a demyelinating disease of the central nervous system (CNS) that affects an estimated 2.3 million people worldwide (1). The symptoms of MS are highly varied but frequently include pain, muscle spasticity, fatigue, inflammation, and depression. These symptoms often lead to reduced physical activity, negatively impact functional mobility, and have a detrimental impact on patients’ quality of life. Although recent years have seen significant advances in disease modifying therapy, none of the current treatments halts or cures MS related symptoms (2). As a consequence, many people with MS (PwMS) look for alternative and complementary therapies such as cannabis.

The cannabis plant contains many biologically active chemicals, including ~60 cannabinoids (3). Cannabidiol (CBD) and Δ9-tetrahydrocannabinol (THC) are typically the most concentrated chemical components of cannabis and believed to primarily drive therapeutic benefit. There is evidence that CBD has a number of beneficial pharmacological effects (4, 5). It is anti-inflammatory, antioxidative, antiemetic, antipsychotic, and neuroprotective. The review of 132 original studies by Bergamaschi et al. (6) describes the safety profile of CBD by highlighting that catalepsy is not induced and physiological parameters (heart rate, blood pressure, and body temperature) are not altered. Moreover, psychomotor and psychological functions are not negatively affected. High doses of up to 1,500 mg per day and chronic use have been repeatedly shown to be well tolerated by humans (6). Additionally, there is also evidence that CBD may reduce the negative psychotropic effects, memory impairment, and appetite stimulation, anxiety and psychotic-like states of THC while enhancing its positive therapeutic actions (7, 8).

Currently, many PwMS utilize cannabis to manage a variety of symptoms. Kindred et al. (9) showed in a web-based survey, which was hosted by the National Multiple Sclerosis Society that 66% of PwMS currently use cannabis for symptom treatment. Furthermore, a study from Canada found that approximately 50% of PwMS would consider the usage of cannabis if the legal status is clear and scientific evidence is available (10). Cannabis is legal in twenty-nine states for the use of specific medical conditions—including MS. Sixteen more states have passed laws that explicitly allow the medical use of CBD. It is suggested that recent increases in the social acceptance of CBD will lead to increases in the number of PwMS using cannabis to treat their symptoms. Anecdotal reports indicate that an increasing number of PwMS use cannabis (medical marijuana) as a supplement to improve their mobility.

Based on the following considerations, it is our opinion that CBD supplementation maybe advisable for PwMS to reduce fatigue, pain, spasticity, and ultimately improve mobility. An overview of the potential impacts of CBD on mobility of PWMS is show in Figure 1.

**Figure 1:**
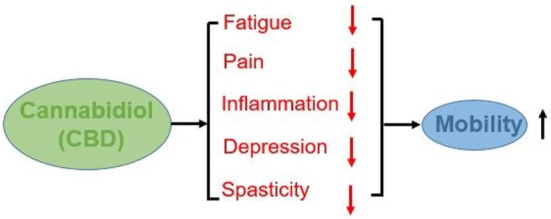
Impacts of CBD on mobility.

## Cannabidiol Reduces Spasticity, Pain, Inflammation, Fatigue, and Depression in PwMS

Despite the common use of and interest in cannabis by people with MS (PwMS), there is very limited empirical data pertaining to its impact on physical mobility. The benefits related to cannabis use in PwMS are still under investigation. However, data indicates that cannabis, with 1:1 or greater CBD:THC ratio, reduces muscle spasticity (11) and pain in PwMS (12). The American Academy of Neurology (13) has highlighted cannabis’ safety profile as well as these benefits. However, there are currently no studies, which investigated the effects of cannabis on mobility in PwMS, some studies have suggested that cannabinoids may exert positive effects on health by decreasing inflammation and decreasing pain (6). Furthermore, inflammation plays an important role in the generation of MS related fatigue (14). Specifically, chronic peripheral inflammation and a resulting overactivity of the vagus nerve are related to fatigue in PwMS (14). There is indirect evidence that reductions in spasticity, pain, and fatigue may result in improvements in the mobility of PwMS (15–17). Furthermore, it is suggested that CBD showed a dose-dependent antidepressant-like effect in the animal model (18). The exact mechanism underlying such activity is still unknown. Depression is an important contributory factor to the observed impaired mobility in PwMS (15). Based on extant evidence we propose that the impact of cannabidiol (CBD) on mobility to be investigated.

## Cannabis Reduces the Usage of Prescription Drugs, Particularly Pharmaceutical Opiods, Benzodiazepines, and Antidepressants

These medications continue to be widely prescribed in the majority of PwMS suffering from pain, spasticity, anxiety, and panic disorders. Common side effects of opioid administration include physical dependence, dizziness, sedation, nausea, vomiting, tolerance, constipation, and respiratory depression. Physical dependence and addiction are clinical concerns that may prevent accurate prescribing and in turn insufficient pain management. Traditional benzodiazepines are associated with sleep disturbances and anterograde amnesia. Another concern with long duration benzodiazepines such as diazepam or flurazepam, is drowsiness and “hangover effect.” Antidepressants can cause a wide range of unpleasant side effects, including nausea, fatigue and drowsiness, blurred vision, dizziness, and anxiety. It is obvious that those drugs delay or even prevent successful physical rehabilitation. A recent epidemiological study by Piper et al. (19) showed that among people that frequently used opioids, over three-quarters (77%) indicated that they reduced their use since they started cannabis. Approximately two-thirds of patients decreased their use of antianxiety (72%), migraine (67%), and sleep (65%) drugs following medical cannabis which significantly exceeded the reduction in antidepressants or alcohol use. Complete or part replacement of these drugs by specific cannabis products should definitely be the long-term goal.

However, objections to the notion that cannabinoids should be used to improve the mobility in PwMS include the following: (1) limited scientific evidence for the effectiveness of cannabis on mobility in PwMS; (2) uncertainty of legal status; (3) social stigmatization from friends, family, and authorities such as employers, landlords, and law enforcement; (4) incidence of dependency; and (5) negative psychoactive effects of cannabis. These objections have some merit and should be taken into consideration. It is important to note that the psychoactive effects of cannabis, such as cognitive impairments, psychosis, and anxiety are due to tetrahydrocannabinol (THC). However, CBD has antipsychotic properties and can also counter several negative side effects of THC. Most PwMS prefer to avoid feeling *high*. Therefore, individuals should seek out strains of cannabis containing equal or higher levels of CBD, compared to THC. Another concern is the risk of *addiction*. It is estimated that ~9% of individuals utilizing cannabis will become dependent on the drug (20). Although a significant risk, this incidence of dependency is significantly lower than that of approved chronic pain management pharmaceuticals (21). Observing for cannabis dependency is suggested for all patients.

### Things to Consider

#### CBD-Drug Interactions

Serious drug interactions have not been seen with CBD in combination with any other drugs.

However, CBD and other plant cannabinoids can potentially interact with many pharmaceuticals. For example, the activity of liver enzymes such as cytochrome P450 is impacted. More than 60 percent of marketed pharmaceuticals are metabolized by this group of enzymes. At high enough dosages, CBD will temporarily deactivate these liver enzymes, thereby altering how a wide range of compounds is metabolized. The exact mechanisms are unknown and more human studies, which monitor CBD-drug interactions are needed (22). PwMS who are taking any prescription medications are strongly advised to consult with a medical professional.

#### Labeling Accuracy of CBD Extracts

A major concern is the often labeling accuracy of CBD extracts. Bonn-Miller et al. (23) found a wide range of CBD concentrations among CBD products purchased online. The tested products contain 26% less CBD than labeled, which could negate any potential clinical benefit. The over labeling of CBD products and that THC was detected (up to 6.43 mg/mL) in 18 of the 84 samples tested suggest that there is a need for federal and state regulatory agencies to take steps to ensure label accuracy of CBD products sold online and in dispensaries.

#### Can a Cannabidiol User Test Positive for Marijuana?

In the CBD products without THC, then a urine test would not yield a positive result for THC metabolites. However, most CBD products contain minimal amounts of THC in CBD. An important aspect in cannabinoid compounds is the entourage effect. The entourage effect means that the compounds in cannabis work more sufficient together than if the compounds are isolated. Therefore, CBD products may contain more cannabis compounds, including THC, to increase the effectiveness of the product (7). Furthermore, often a study by Merrick et al. (24) is cited which showed that CBD could be converted into THC after prolonged exposure to “simulated” gastric acid. However, there is no scientific evidence that this reaction occurs *in vivo* in humans (25). If someone is using a CBD product and needs to undergo urine drug tests, lab reports should be requested and examined to ensure that the CBD product contain exactly what is expecting and on the label.

It is clear that more research is needed. However, because of the safety of CBD and if the concerns listed above are accounted, we are in the opinion that we already have some good reasons to believe that CBD enriched cannabis is useful to improve the mobility of PwMS.

## Author Contributions

TR and JS contributed to drafting the article and revising it critically for important intellectual content. All the authors approved the final version of the manuscript.

## Conflict of Interest Statement

The authors declare that the research was conducted in the absence of any commercial or financial relationships that could be construed as a potential conflict of interest.
